# School climate and academic burnout in medical students: a moderated mediation model of collective self-esteem and psychological capital

**DOI:** 10.1186/s40359-023-01121-6

**Published:** 2023-03-22

**Authors:** Wanwan Yu, Wenjun Yao, Ming Chen, Hongqing Zhu, Jing Yan

**Affiliations:** 1 The Second Clinical Medical School, Anhui Medical University, Hefei, 230032 China; 2 Department of Radiology, The Second Affiliated Hospital of Anhui Medical University, Hefei, 230601 China; 3 School of Health Service Management, Anhui Medical University, Hefei, 30032 China

**Keywords:** School climate, Collective self-esteem, Psychological capital, Academic burnout, Medical students

## Abstract

**Background:**

The study burnout of medical students is more and more serious, which directly affects the study style of university and the learning quality of students. This has aroused the high attention of researchers and universities. This study aimed to explore the mechanism of the influence of school climate on academic burnout among medical students in Chinese cultural context.

**Methods:**

2411 medical students (50.52% female; mean age = 19.55, *SD* = 1.41, rang = 17–24 years) were investigated with psychological environment questionnaire, collective self-esteem scale, psychological capital scale and academic burnout scale. The data were analyzed by using a moderated mediation model with SPSS and the Process 4.0 macro.

**Results:**

The results revealed that: (1) school climate had a significant negative predictive effect on academic burnout among medical students controlling for gender, grade and age (*B* = -0.40, *p* < 0.001). (2) Collective self-esteem played a partial mediating role in school climate and academic burnout (indirect effect = -0.28, 95% CI = [-0.32,-0.25], accounting for 52.83%). (3) The first and second half of the indirect effect of school climate on medical students’ academic burnout were moderated by psychological capital (*B* = 0.03, *p* < 0.01; *B* = -0.09, *p* < 0.001).High level of psychological capital can enhance the link between school climate and collective self-esteem as well as the link between self-esteem and academic burnout.

**Conclusion:**

Creating a good school atmosphere and improving the level of collective self-esteem and psychological capital are beneficial to improve the academic burnout of medical students.

## Introduction

Academic burnout is a negative attitude and behaviour of students who are bored with learning due to pressure or lack of interest in learning [1]. Its negative effects are mainly reflected in physical and mental health (e.g. insomnia, weakness), emotional adaptation (e.g. anxiety, depression) and behaviour (e.g. aggression, dropping out of school) [2–4]. Medical students are more prone to academic burnout due to their long training cycles, course content and heavy study load as a reservoir of healthcare professionals [5]. A meta-analysis study showed that the detection rate of academic burnout among medical students was about 44.2% [6]. Therefore, it is important to explore the factors influencing academic burnout among medical students and its mechanisms of action to promote the quality of learning and positive development of medical students.

### School climate and academic burnout

Schools are important micro-systems that influence the growth and development of individuals in addition to the family [7]. They are not only places where individuals learn and develop cognitively, but are also important contextual factors for the formation of positive social relationships and for their emotional and behavioral development [8]. As a result, a growing body of research has focused on the impact of school climate on the physical and psychological development of individuals [9–11]. School climate, also known as the school psychological environment, refers to the relatively persistent and stable features of the environment that students experience and influence their behaviour [12], including the norms, goals, values, interpersonal relationships, teaching practices and organizational structures of the school environment [13]. Numerous studies have shown that school climate is strongly associated with healthy adolescent development, with the more positive the perceived school climate, the less suicidal ideation, depression, bullying, etc. [14, 15]. According to the Stage-Environment Matching Theory, when the school climate meets the developmental needs of students, it strengthens the connection between individuals and the school which could promote good development; on the contrary, when the school climate does not meet the developmental needs of students, they are prone to psychological and behavioral problems [16, 17]. A positive school climate in terms of teacher-student relationships, peer relationships and student autonomy can lead to a strong attachment to the school in which students are studying, which is an important protective factor against problematic behaviour [18] and can push motivation to learn and less likely to develop academic burnout [19]. Empirical studies have also found that school climate is significantly and negatively associated with academic burnout in primary school students [20]. Previous study on the relationship between school climate and academic burnout has focused on primary and secondary school students, and less on medical students. However, there is an inherent consistency between university campuses and primary and secondary school campuses, mainly in terms of school norms and discipline, teacher-student relationships, peer relationships, and physical environment, which are all important components of school climate [21]. Accordingly, this study proposes hypothesis 1: School climate negatively predicts academic burnout among medical students.

Although the relationship between school climate and academic burnout has been partially verified by researchers, the exact mechanisms of the relationship are still largely unclear. Therefore, the question of how school climate “influences” medical student burnout needs to be further explored.

### The mediating role of collective self-esteem

The ego self-system process model suggests that external environmental resources influence developmental outcomes through an individual’s internal self-system [22]. Therefore, the influence of school climate on medical student burnout may be mediated by collective self-esteem. Self-esteem, as an important component of the ego system, includes both individual and collective self-esteem [23] and is an important protective resource for mental health [24]. The former is the overall positive evaluation and acceptance of individuals to themselves [25, 26], while the latter is the individual’s evaluation and perception of the value of the group he or she belongs to, which emphasizes a sense of collective value, respect and belonging [27]. In the context of Chinese collectivist culture, self-esteem, with its strong collective and social overtones, has also become a focus of research [28, 29]. Existing studies also indirectly support the mediating path of school climate - collective self-esteem - academic burnout. On the one hand, collective self-esteem helps to reduce academic burnout. Self-categorization theory suggests that the pursuit of self-improvement and self-esteem is one of the most basic motivations of people [30]. Since in-group evaluations are essentially evaluations of the self, when individuals identify with the group to which they belong, they would strive to enhance or protect the prestige and status of their group in relation to other groups, producing more adaptive outcomes [31]. Empirical studies have also shown that collective self-esteem significantly and negatively predicts academic burnout, individuals with low collective self-esteem tend to lack good motivation to learn and thus exhibiting more academic burnout [32, 33]. On the other hand, collective self-esteem is influenced by school climate. According to sociometric theory, a positive school climate, typically reflecting a supportive environment [34], enhances psychological and socio-emotional functioning and raises one’s self-esteem [35–37]. Empirical studies have also shown that school climate can be a significant positive predictor of collective self-esteem [38–40]. When students perceive that they are accepted and supported by important people (e.g. teachers, classmates), they also have a stronger sense of belonging and identification with school and show higher levels of collective self-esteem [41, 42]; conversely, a negative school climate decreases students’ overall evaluation of school and shows lower levels of collective self-esteem [43]. Accordingly, this study proposes hypothesis 2: collective self-esteem may mediate the relationship between school climate and academic burnout.

### The moderating role of Psychological capital

Although collective self-esteem may mediate the relationship between school climate and academic burnout, there may still be moderating variables, i.e. the process by which school climate influences academic burnout may be moderated by other factors. With the rise of positive psychology, the additive role of psychological capital on individual psychology and behaviour has received increasing attention from researchers [44–46]. According to mental resilience research, exposure to situational risk does not imply poor development of mental health, and certain positive qualities (e.g. psychological capital) help individuals overcome the adverse effects of severe adversity [47, 48]. Thus, psychological capital may play a moderating role in the process of school climate affecting academic burnout. Psychological capital refers to a positive psychological state that individuals demonstrate as they grow and develop, and includes four core elements: resilience (the ability to recover and grow positively from adversity and setbacks), optimism (positive beliefs about the present and future), hope (a positive state of motivation to strive to achieve goals in multiple ways) and self-efficacy (the belief in one’s ability to succeed when faced with challenging tasks) [49], which is an important protective factor for individual psychological well-being and facilitates resistance to the adverse effects of external stressors such as academic burnout [50, 51]. Previous research has shown that psychological capital is negatively associated with academic burnout in nursing students, increasing engagement in learning and reducing academic burnout [52]. All elements of psychological capital also positively predict collective self-esteem, high psychological capital has positive and effective coping strategies, which exhibiting higher levels of collective self-esteem [53]. Self-depletion theory suggests that human psychological resources are limited, and although individuals can mobilize internal resources to cope with various external risks, they are not replenished in time and are prone to negative consequences [54]. As a positive psychological quality, individuals with high psychological capital can cope with the negative effects of a negative school climate [55], and enhance the positive effects of a positive school climate on cognition, mood and behavior [56]. The individual-environment interaction model states that individual development is the result of the interaction between individual factors and the environment in which the individual lives [57]. This means that academic burnout and collective self-esteem are not only closely related to environmental factors (school climate), but may also be influenced by individual factors (psychological capital). This suggests that psychological capital may regulate the relationship between school climate and collective self-esteem or academic burnout.

Secondly, psychological capital and collective self-esteem, as protective factors, may work together to reduce academic burnout. Psychological capital has been found to enhance the positive impact of protective factors on individual school adjustment [56]. According to the protective factor-protective factor model, different protective factors may interact with each other to influence developmental outcomes [58]. Researchers have summarized two different hypotheses: the facilitation hypothesis and the exclusion hypothesis [59, 60]. According to the facilitation hypothesis, the effect of school climate on collective self-esteem or academic burnout and collective self-esteem on academic burnout is stronger as the level of psychological capital increases [61]. According to the exclusion hypothesis, as the level of psychological capital increases, the effect of school climate on collective self-esteem or academic burnout and collective self-esteem on academic burnout decreases [62]. At present, there has been on research point out the moderating mode of psychological capital in the relationship between school climate and academic burnout (direct and indirect pathways). Accordingly, this study only hypothesized that psychological capital may play a moderating role in the influence of school climate on academic burnout through collective self-esteem (Hypothesis 3), without making specific hypotheses about the specific moderating patterns.

**Fig. 1:**
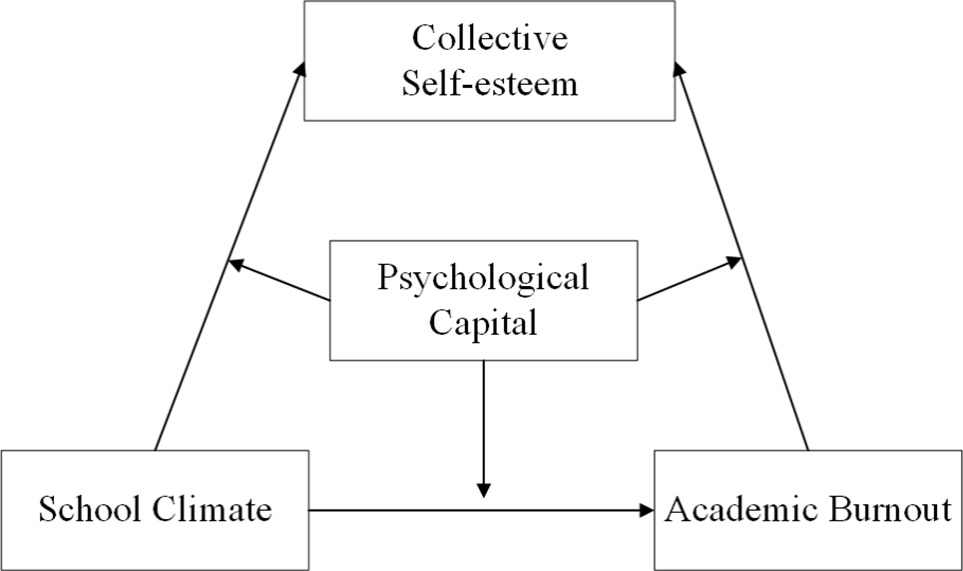
Hypothetical model

In summary, this study constructs a moderated mediation model based on the Stage-Environment Matching Theory and the Protective Factor-Protective Factor Model (see Fig. 1 for the hypothetical model) to examine the mediating role of collective self-esteem in the relationship between school climate and medical students’ academic burnout, and the role of psychological capital in this mediating role. The model is designed to test the role of collective self-esteem in mediating the relationship between school climate and medical student burnout, and the moderating role of psychological capital in this mediating process, in order to provide guidance for the prevention and reduction of medical student burnout.

## Methods

### Participants

A cluster random sampling method was used to select 2,600 medical students from seven medical colleges and universities in Anhui Province as the research objects. 189 invalid questionnaires with missing values and test results outside ± 3 standard deviations were excluded [63]. Finally, 2411 valid questionnaires were obtained, and the effective recovery rate was 92.73%. Among them, there were 1193 boys (49.48%), 1218 girls (50.52%); 641 freshmen (26.59%), 673 sophomores (27.91%), 585 juniors (24.26%), and 512 seniors (21.24%). The subjects were between 17 and 24 years old, with an average age of 19.55 years (SD = 1.41 years).

## Measures

### School climate

The “School Psychological Environment Questionnaire” (SPEQ) developed by Xu and Zhong [64] was used to measure the subjects’ perceived school climate, and has been validated to have good reliability and validity [65]. The questionnaire has a total of 30 items, including 7 reverse questions, which is composed of six dimensions: teacher-student relationship (e.g., “teacher cares about me.”), classmate relationship (e.g.,“classmates care about each other.”), collective activities (e.g., “I participate in group activities.”), professional development (e.g. “I learned about possible careers in the future.”), resources (e.g., “The resources in the school do not meet my needs.”), and institutions and order (e.g., “The rules and regulations of the department are helpful for the development of students.”). A 5-point Likert scale was used, with 1–5 points for “Never” to “always” respectively. After reverse scoring, the average score for all items was calculated, with higher scores indicating more positive school climate perceived. In this study, the Cronbach’s α coefficient of this questionnaire was 0.93.

### Collective self-esteem

The collective self-esteem level of the subjects was measured using the “collective self-esteem scale” (CSES) developed by Luhtanen and Crocker [66], and has been validated to have good reliability and validity [67]. The scale has 16 items, including 8 reverse questions, which is composed of four dimensions: membership self-esteem (e.g.,“I am a valuable member of the class collective.“), private collective self-esteem (e.g., “I feel good about the school where I belong.”), public collective self-esteem (e.g., “generally, others respect our school.”), identity importance (e.g., “generally, the identity of school group members is an important part of my self-image.”). A 7-point Likert scale was used, with 1–7 points for “very inconsistent” to “very consistent” respectively. After the reverse scoring, the average score was calculated for all items, with higher scores indicating higher levels of collective self-esteem. In this study, the Cronbach’s α coefficient of this scale was 0.87.

### Psychological capital

The “Positive Psychological Capital Questionnaire” (PPQ) developed by Zhang et al. [68] was used to measure the psychological capital level of the subjects, and has been validated to have good reliability and validity [69]. The questionnaire consists of 26 items, consisting of four dimensions of optimism (e.g., “I always see the bright side of things.“), hope (e.g., “I pursue my goals with confidence.“), self-efficacy (e.g., “I can always do well Completion of tasks.”) and resilience (e.g., “When I encounter setbacks, I can quickly recover.”).A 7-point scale was used, with 1–7 points for “strongly disagree” to “strongly agree” respectively. Calculate the average score of all items and higher scores indicate higher levels of psychological capital. In this study, the Cronbach’s α coefficient of this questionnaire was 0.94.

### Academic burnout

The academic burnout scale (LBS) developed by Lian et al. [1], and has been validated to have good reliability and validity [70]. The scale has 20 items in total, including 8 reverse questions, which are composed of three dimensions: low mood(e.g., “I am tired of studying.“), low sense of accomplishment(e.g.,“I am energetic when studying.“) and improper behavior(e.g., “only I only read it when I take the test.”). A 7-point Likert scale is used, with 1–7 points for"very inconsistent” to “very consistent”. After reverse scoring, the average score for all items is calculated, with higher scores indicating higher levels of academic burnout. In this study, the Cronbach’s α coefficient of this scale was 0.89.

### Procedures and statistical analysis

After obtaining the informed consent of the subjects, group testing was conducted in a class as a unit. The main test was held by a strictly trained graduate student majoring in psychology, who explained the instructions in detail to the subjects and emphasized that the content was strictly confidential and answered anonymously according to their actual situation. The test time took 12 min in total and all the questionnaires were collected on the spot.SPSS 25.0 and PCOCESS3.3 developed by Hayes were used for data processing and analysis [71].

## Results

### Common method bias and multicollinearity test

Restricted by objective conditions, this study only used the method of self-report by the subjects to collect data, and the results may be affected by common method bias. According to the suggestions of Podsakoff et al. [72], control is carried out in terms of procedures, such as using an anonymous method for testing and using reverse questions for some items. After data collection was completed, HarmanOne-factor test was used to test for common method bias. The results showed that a total of 13 factors with eigenvalues greater than 1were extracted,and the unrotated variation of the first factor was 28.91%, which was lower than the critical standard of 40%. At the same time, the variance inflation factor values of all predictors are between 2.02 and 2.09 (less than 5 means there is no collinearity), and the tolerance is between 0.45 and 0.50 (more than 0.1 means there is no collinearity) [73]. Therefore, the influence of common method bias and multicollinearity on the results of this study is basically excluded.

### Descriptive statistics and correlation analysis

Table 1 lists the mean, standard deviation and correlation matrix for each variable. The results showed that academic burnout was significantly negatively correlated with school psychological environment (*r* = -0.54, *p* < 0.001), collective self-esteem (*r* = -0.60, *p* < 0.001) and psychological capital (*r* = -0.69, *p* < 0.001); School psychological environment was significantly positively correlated with collective self-esteem (*r* = 0.68, *p* < 0.001) and psychological capital (*r* = 0.63, *p* < 0.001); collective self-esteem was significantly positively correlated with psychological capital (*r* = 0.67, *p* < 0.001). Considering that gender, grade, and age were significantly correlated with the main research variables, the three were included as control variables in the subsequent analysis to improve the accuracy of the model.

**Table 1:** Mean, standard deviation and correlation matrix of each variable

Variable	1	2	3	4	5	6	7	8	9
1. Gender ^a^	1								
2. Sophomore^b^	-0.01	1							
3. Junior ^b^	0.01	-0.35^***^	1						
4. Senior ^b^	0.07^***^	-0.32^***^	-0.29^***^	1					
5. Age	0.02	-0.18^***^	0.30^***^	0.57^***^	1				
6. School climate	-0.002	-0.06^***^	-0.07^***^	-0.12^***^	-0.19^***^	1			
7. Collective Self-Esteem	0.06^***^	-0.09^***^	-0.13^***^	-0.07^***^	-0.20^***^	0.68^***^	1		
8. Psychological Capital	-0.05^*^	-0.07^***^	-0.10^***^	-0.04	-0.14^***^	0.63^***^	0.67^***^	1	
9. Academic burnout	-0.01	0.10^***^	0.07^***^	-0.004	0.07^***^	-0.54^***^	-0.60^***^	-0.69^***^	1
Mean	0.51	0.28	0.24	0.21	19.55	3.41	4.77	4.77	2.71
Standard deviation	0.50	0.45	0.43	0.41	1.41	0.52	0.79	0.83	0.54

Note: ***n*** = 2411. ^a^ gender is a dummy variable, boys = 0, girls = 1, and the mean represents the proportion of *g*irls. ^b^grad*e* is a dummy variable, with freshman as the reference category, sophomore, junior and senior are relative to this category forms 3 dummy variables, the mean values of which represent the percentage of the population in that grade to the total population. ***p < 0.001, **p < 0.01, *p < 0.05, the same below

### Moderated mediation test

First, using Model4 in the SPSS macro program PROCESS (Model4 is a simple mediation model) to test the mediating effect of collective self-esteem in th*e* relationship between school psychological environment and academic burnout under the control of gender, grade and age. The results are shown in Table 2, the school psychological environment significantly negatively predicted academic burnout (*B* = − 0.40, *p* < 0.001), and significantly positively predicted collective self-esteem (*B* = 0.65, *p* < 0.001); When predicting academic burnout at the same time, school psychological environment can still significantly negatively predict academic burnout (*B* = -0.25, *p* < 0.001), and collective self-esteem significantly negatively predicts academic burnout (*B* = -0.44, *p* < 0.001). Based on the bias-corrected percentile Bootstrap method, it was further found that collective self-esteem played a partial mediating role between the school psychological environment and academic burnout, and its 95% CI was [-0.32, -0.25]. The mediating effect (-0.28) accounted for 52.83% of the total effect (-0.53).

**Table 2:** Mediation model test of collective self-esteem

Predictor variable	Equation 1 (efficacy criterion: academic burnout)				Equation 2 (efficacy criterion: collective self-esteem)				Equation 3 (efficacy criterion: academic burnout)		
	*B*	*SE*	*95% CI*		*B*	*SE*	*95% CI*		*B*	*SE*	*95% CI*
School climate	-0.53^***^	0.02	[-0.57,-0.50]		0.65^***^	0.02	[0.62,0.68]		-0.25^***^	0.02	[-0.29,-0.20]
collective self-esteem									-0.44^***^	0.02	[-0.48,-0.39]
Gender ^a^	-0.02	0.03	[-0.09,0.05]		0.13^***^	0.03	[0.07,0.19]		0.04	0.03	[-0.03,0.10]
Sophomore ^b^	0.20^**^	0.05	[0.09,0.03]		-0.28^***^	0.04	[-0.37,-0.19]		0.07	0.05	[-0.02,0.17]
Junior ^b^	0.16^*^	0.07	[0.03,0.20]		-0.32^***^	0.06	[-0.44,-0.21]		0.02	0.06	[-0.10,0.14]
Senior ^b^	0.01	0.08	[-0.16,0.15]		-0.14^*^	0.07	[-0.27,-0.001]		-0.07	0.07	[-0.21,0.08]
Age	-0.03	0.03	[-0.09,0.03]		-0.03	0.02	[-0.08,0.01]		-0.04	0.03	[-0.10,0.01]
*R* ^*2*^	0.30				0.49				0.39		
*F*	168.66^***^				378.50^***^				223.83^***^		

Note: All variables in the model are brought into the regression equation after standardization, the same below

Secondly, Model59 in SPSS macro program PROCESS (adjusting the first half path,the second half path and the direct path, which is consistent with the hypothetical model)was used to test the moderating effect of psychological capital under the condition of controlling gender, grade and age. The results are shown in Table 3. After adding psychological capital, the interaction term between school psychological environment and psychological capital significantly positively predicted collective self-esteem (*B* = 0.03, *p* < 0.01), indicating that psychological capital modulates the relationship between school psychological environment and collective self-esteem; The interaction item of collective self-esteem and psychological capital significantly negatively predicted academic burnout (*B* = -0.09, *p* < 0.001), indicating that psychological capital moderates the relationship between collective self-esteem and academic burnout; while the interaction term between school psychological environment and psychological capital had no significant predictive effect on academic burnout (*B* = -0.02, *p* > 0.05), indicating that psychological capital could not moderate the relationship between school psychological environment and academic burnout. In conclusion, school psychological environment, collective self-esteem, psychological capital, and academic burnout constitute a moderated mediating model. Specifically, school psychological environment influences the first half path of academic burnout through collective self-esteem, and the second half path is regulated by psychological capital.

**Table 3:** Moderated mediation model test of school climate on academic burnout

Predictor variable	Equation 1 (efficacy criterion: collective self-esteem)				Equation 2 (efficacy criterion: academic burnout)		
	*B*	*SE*	*95% CI*		*B*	*SE*	*95% CI*
School climate	0.40^***^	0.02	[0.36,0.43]		-0.06^***^	0.02	[-0.10,-0.02]
Psychological capital	0.38^***^	0.02	[0.35,0.42]		-0.50^***^	0.02	[-0.54,-0.46]
School climate × Psychological capital	0.03^**^	0.01	[0.01,0.05]		-0.02	0.02	[-0.05,0.01]
Collective self-esteem					-0.19^***^	0.02	[-0.24,-0.15]
Collective self-esteem × Psychological capital					-0.09^***^	0.02	[-0.13,-0.06]
Gender^a^	0.17^***^	0.03	[0.12,0.23]		-0.07^*^	0.03	[-0.12,-0.01]
Sophomore ^b^	-0.23^***^	0.04	[-0.31,-0.15]		0.07	0.04	[-0.02,0.15]
Junior ^b^	-0.28^***^	0.05	[-0.38,-0.17]		0.01	0.05	[-0.09,0.12]
Senior ^b^	-0.16^**^	0.06	[-0.29,-0.04]		-0.03	0.07	[-0.16,0.09]
Age	-0.02	0.02	[-0.06,0.02]		-0.05	0.02	[-0.09,0.00]
*R* ^*2*^	0.58				0.53		
*F*	406.51^***^				275.27^***^		

**Fig. 2:**
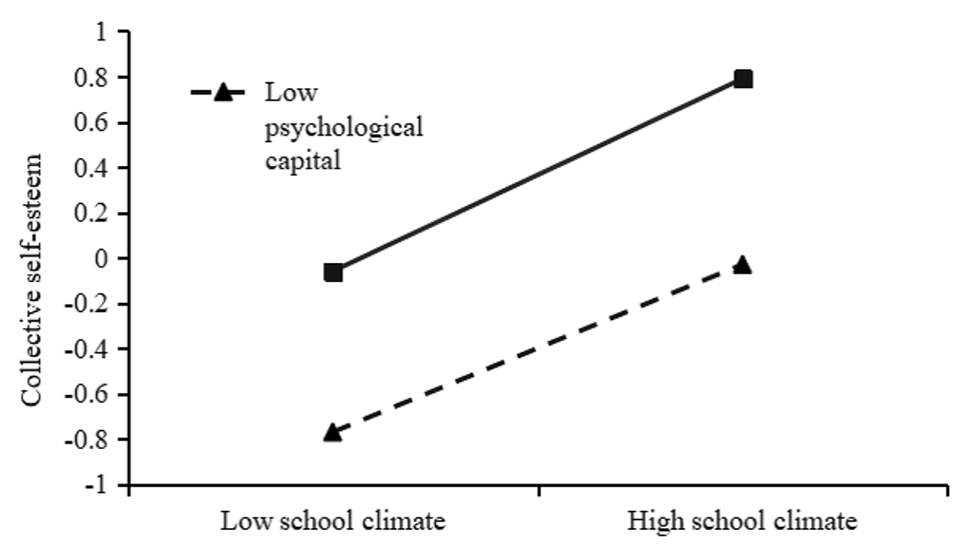
The interaction between school climate and psychological capital on collective self-esteem

**Fig. 3:**
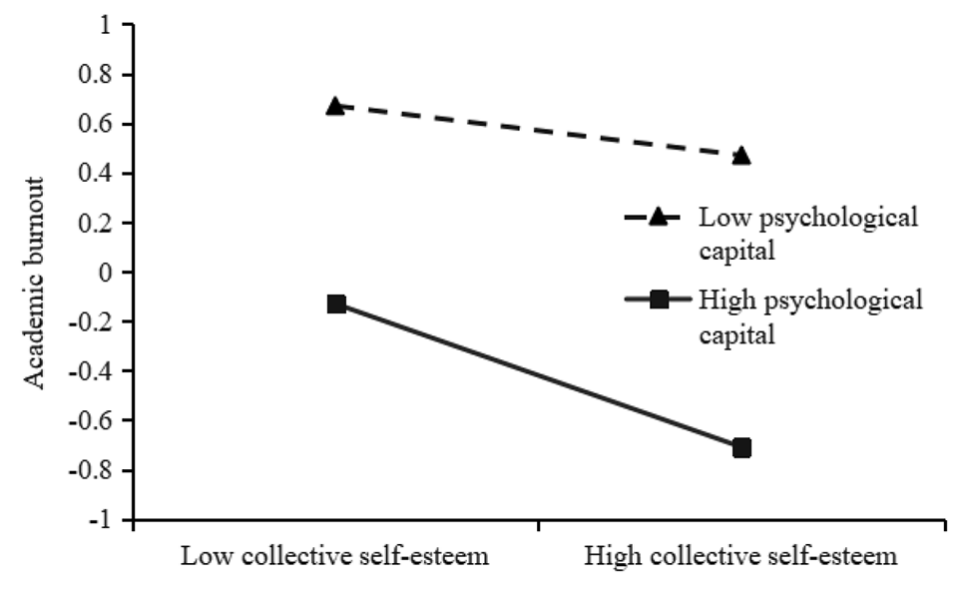
The interaction between collective self-esteem and psychological capital on academic burnout

In order to better explain the moderated mediation model, psychological capital is divided into high group and low group according to the mean plus or minus one standard deviation. simple slope analysis is performed and a simple effect analysis graph is drawn. As shown in Fig. 2, when the individual’s psychological capital level is low, the school psychological environment significantly positively predicts collective self-esteem (*B*_simple_ = 0.37, *t* = 16.91, *p* < 0.001); when the individual’s psychological capital level is high, the school Mental environment still significantly positively predicted collective self-esteem and increased predictive power (*B*_simple_ = 0.43, *t* = 22.44, *p* < 0.001). The results show that with the increase of psychological capital level, the predictive effect of school psychological environment on collective self-esteem is enhanced. It can be seen from Fig. 3 that when the individual’s psychological capital level is low, collective self-esteem significantly negatively predicts academic burnout (*B*_simple_ = -0.10, *t* = -3.29, *p* < 0.01); when the individual’s psychological capital level is high, school Mental environment still significantly negatively predicted collective self-esteem and increased predictive power (*B*_simple_ = -0.29, *t* = -10.77, *p* < 0.001). The results show that with the increase of psychological capital level, the predictive effect of collective self-esteem on academic burnout is enhanced.

## Discussion

This study found that school climate significantly and negatively predicted medical students’ academic burnout, confirming research hypothesis 1. This suggests that a positive school climate is an important protective factor for medical students’ academic burnout, reducing the likelihood of academic burnout and providing good environmental conditions for medical students’ learning, which is consistent with previous research [18, 20]. The results also support the stage-environment match theory, which states that matching the school environment with student needs promotes more positive outcomes [16]. Self-determination theory suggests that autonomy, relationship and competence needs are basic psychological needs of individuals and are motivational in nature [74]. Medical students are largely mature in their physical and mental development and desire to have their basic needs met. Good teacher-student relationships, peer relationships, fair rules and order on campus can meet these needs, making students more inclined to align with the school’s goals and values, motivating intrinsic learning and actively seek academic progress [75]; Conversely, when school fail to meet this psychological need, students may turn to other environments for satisfaction, such as online games [76], which in the long run may lead to problems such as lack of interest in learning and reduced motivation to learn, which may lead to truancy or a sense of alienation from learning in turn.

Moreover, this study also found that school climate can indirectly influence medical students’ academic burnout through collective self-esteem, confirming hypothesis 2. This result also confirms the self-system process model [22], which suggests that collective self-esteem plays a key role in individual adaptation to the external environment and is a proximal factor in school climate influencing academic burnout. Firstly, positive campus characteristics such as good teacher-student relationships, sound infrastructures and fair systems promote a higher sense of belonging to the school, making students feel part of the school, more aware of their importance and value in their group, hold more positive evaluations of the school and show higher levels of collective self-esteem [65]. Secondly, anxiety is an important emotional manifestation of academic burnout, and self-esteem is an important force in reducing anxiety [26, 77]. Individuals with high collective self-esteem are more likely to have a positive emotional experience of school, maintain a good emotional state even when suffering from chronic academic stress, seek social support to alleviate anxiety, and engage in learning with a positive attitude, thereby reducing academic burnout [78]. The rationale for collective self-esteem as a mediating variable is also supported by the perspective of social control theory. The theory emphasizes that the emotional connection between the individual and the school is a protective factor for individual development, and that this connection causes the individual to strive to align with social expectations, thereby avoiding negative consequences [10]. A positive school climate can lead to students developing a positive emotional connection with school, enabling them to control their own behaviour, move towards the academic goals expected by the school, and become actively engaged in learning activities, thereby reducing academic burnout [79]; conversely, when students lack this emotional connection with school, they can become negative about school activities, even if they believe that academics are important to them. In contrast, when students lack this emotional connection to school, they may become resistant to school activities, even if they consider it important, and gradually lose interest and enthusiasm for learning, which can be detrimental to academic achievement [80].

Additionally, this study also found that psychological capital moderates the mediating process of “school climate → collective self-esteem → academic burnout”, in that the first and second halves of the mediating chain are moderated by psychological capital, which partially confirms research hypothesis 3. On the one hand, psychological capital moderates the relationship between school climate and medical students’ collective self-esteem. School climate moderates the relationship between school climate and the collective self-esteem of medical students, which is more influential on the collective self-esteem of medical students with high psychological capital than those with low psychological capital. This moderating model is consistent with the facilitation, rather than exclusion, hypothesis of the “protective factor-protective factor” model, which suggests that psychological capital, as a positive psychological quality, can mobilize its own resources to enhance self-perceptions in a positive school environment [81], and supports the individual-environment interaction model [57]. Resource conservation theory suggests that students become relatively vulnerable when they are under-resourced and are likely to experience greater psychological stress and negative emotions; when they are well resourced they have better coping skills and a greater sense of self-worth and competence [82, 83]. Medical students with high psychological capital possess positive psychological qualities such as self-efficacy, hope, resilience and optimism, and are more sensitive to the positive components of the school climate, and are able to adopt cognitive strategies to adjust their mindset without wavering in their positive perceptions and evaluations of school, even when experiencing stressful events in school life [84].

On the other hand, psychological capital moderates the relationship between collective self-esteem and academic burnout among medical students. Collective self-esteem had a greater impact on academic burnout among medical students with high psychological capital than low psychological capital. This moderating pattern is also consistent with the facilitation hypothesis of the “protective factor-protective factor” model. High psychological capital is closely related to positive emotions [85]. According to the extended construct of positive emotion, individuals with high psychological capital have more flexible cognitive and behavioral patterns and are more likely to receive energy from external sources in response to external risks, whereas individuals with low psychological capital are more vulnerable to external risks and have more difficulty replenishing their energy after attrition [86]. It is evident that high psychological capital medical students, even if they lack an emotional connection to school, compensate for or counteract this negative impact through the various internal and external resources they have, better allocating their attention to their studies and exhibiting lower levels of academic burnout. It is worth noting that psychological capital did not moderate the effect of school climate on academic burnout in medical students, which is inconsistent with previous hypotheses. Further evidence suggests that a good school climate is an important protective factor for individuals’ positive psychological, behavioral development and that their academic burnout is protected by the protective effect of a good school climate regardless of their level of psychological capital [54]. Therefore, to promote good academic adjustment in medical students, it is important to focus not only on the role of internal factors (core self-assessment) but also on the role of external environmental factors (school climate).

## Limitations and future directions

This study reveals the internal mechanism of school climate affecting medical students’ academic burnout, and has a certain reference value for improving school climate and reducing academic burnout. On the one hand, the current study suggests that school managers and educators should pay attention to the role of school climate in academic development, and strive to create a good school climate, which can be considered from the aspects of teacher-student relationship, classmate relationship, system and order, etc. On the other hand, under the circumstance that the school climate cannot be improved in the short term, it can promote the positive academic development by cultivating and improving students’ collective self-esteem and psychological capital level.

Additionally, the current study also has certain limitations that need to be improved in future studies. First, this study uses a cross-sectional study, which is difficult to reveal the causal relationship between variables. Future studies can use follow-up studies to further verify. Secondly, the data of the study comes from students’ self-reports, which may be affected by the social approval effect. Future research may consider adding more objective measurement methods such as experimental methods. Finally, whether the research results can be generalized to other groups needs to be further tested. The research may consider adding more groups (such as primary and secondary school students) to be tested.

## Conclusion

This study constructs a moderated mediation model to explore the process by which school climate acts on academic burnout in medical students. It enriches the theoretical framework of stage-environment fit and provides practical implications for the development of positive psychological resources. Positive school climate can mitigate medical students’ academic burnout levels through collective self-esteem levels, and the relationship between school climate and collective self-esteem and between collective self-esteem and academic burnout is moderated by psychological capital. Therefore, creating a good school climate and improving the level of collective self-esteem and psychological capital is conducive to improving the academic burnout of medical students.

## Data Availability

The datasets generated during and analyzed during the current study are available from the corresponding author on reasonable request.
